# Cellular apoptosis: An alternative mechanism of action for caspofungin against *Candida glabrata*

**DOI:** 10.18502/cmm.5.2.1155

**Published:** 2019-06

**Authors:** Parisa Aryamloo, Hossein Asgarian-Omran, Narges Aslani, Hadi Hossein-Nataj, Tahereh Shokohi, Hamid Badali, Mojtaba Nabili, Atefeh Abdollahi Gohar, Maryam Moazeni

**Affiliations:** 1 Student Research Committee, Mazandaran University of Medical Sciences, Sari, Iran; 2 Department of Immunology, School of Medicine, Mazandaran University of Medical Sciences, Sari, Iran; 3 Immunogenetic Research Center, School of Medicine, Mazandaran University of Medical Sciences, Sari, Iran; 4 Infectious and Tropical Diseases Research Center, Tabriz University of Medical Sciences, Tabriz, Iran; 5 Invasive Fungi Research Center, Mazandaran University of Medical Sciences, Sari, Iran; 6 Department of Medical Mycology, School of Medicine, Mazandaran University of Medical Sciences, Sari, Iran; 7 Department of Medical Laboratory Sciences, Sari Branch, Islamic Azad University, Sari, Iran

**Keywords:** Candida glabrata, Caspofungin, Flow cytometry, MCA1, NUC1

## Abstract

**Background and Purpose:**

Although the mechanism of action for echinocandins is known, the physiological mechanisms by which these antifungal agents cause cell death via the classical apoptotic pathways are not well-defined yet. Regarding this, the present study aimed to evaluate the mechanisms of caspofungin-induced* Candida glabrata* cell death.

**Materials and Methods:**

For the purpose of the study, the minimum inhibitory concentration (MIC) of caspofungin against *C. glabrata* (ATCC 90030) was determined using the broth microdilution reference method (CLSI M27-A2 and M27-S4). The annexin V and propidium iodide staining was performed to determine the way through which caspofungin acts against *C. glabrata* (i.e., through the induction of apoptosis and/or necrosis). Additionally, the possible effect of caspofungin on inducing the expression of two apoptotic genes, namely *MCA1* and *NUC,* was studied using the real-time polymerase chain reaction assay.

**Results:**

According to the obtained MIC value (0.5 µg/mL), *C. glabrata,* exposed to 0.25, 0.5, and 1 µg/mL of caspofungin, exhibited the features of late apoptosis/necrosis after 18 h of incubation. Furthermore, the use of 0.25, 0.5, and 1 µg/ml caspofungin induced apoptosis (early/late) in 14.67%, 17.04%, and 15.89% of the cells, respectively. The results showed a significant difference between the percentages of early-apoptotic cells at the three concentrations (*P<0.05*). In addition, the rate of necrosis was significantly greater than that of apoptosis in response to caspofungin. Accordingly, necrosis occurred in 71.26%, 71.26%, and 61.26% of the cells at the caspofungin concentrations of 0.25, 0.5, and 1 µg/mL, respectively (*P*<*0.05*). The analysis of the data in the REST software demonstrated a significant increase in the expression of *MCA1 *and *NUC1 *genes (*P<0.05*).

**Conclusion:**

As the findings of the present study indicated, caspofungin promoted both necrosis and apoptosis of *C. glabrata *cells at concentrations higher than or equal to the MIC value.

## Introduction

Echinocandins are the newest class of antifungal agents, which inhibit the synthesis of fungal cell wall through the noncompetitive inhibition of the Fks subunits of (1,3)- β-d-glucan synthase [1, 2]. Currently, three echinocandins, including caspofungin, micafungin, and anidulafungin, have been approved for clinical applications [3-6]. The caspofungin is the most widely used echinocandin in clinical settings, which has fungicidal activity against the majority of *Candida* species. Nevertheless, *C. parapsilosis* and *C. guilliermondii* are relatively insensitive to caspofungin [7, 8]. Resistance to echinocandins has also increased significantly in *C. glabrata* with the expanded use of these agents in therapy. Susceptibility testing on 1,380 isolates of *C. glabrata* collected within 2008-2013 showed that 3.3% of the isolates were resistant to caspofungin [9]. 

Resistance to echinocandins in *C. glabrata* and different *Candida* species is explained by the occurrence of mutations in the genes encoding glucan synthases (e.g., *FKS1* and* FKS2*) [10]. Therefore, the development of more effective antifungal agents directly depends on understanding their mechanism of action and the basis of cell death decisions in fungi. Although the spectrum of activity for echinocandins is known, the physiological mechanisms by which these antifungal drugs cause cell death via the classical apoptotic pathways are not identified well. 

Apoptosis is a conserved cell biochemical process of cell death that plays a key role in normal development. Apoptotic cells are characterized by a specific series of morphological and biochemical properties that set apoptosis apart from the accidental cell death and are observed in eukaryotes and prokaryotes [11, 12]. The principal morphological feature of apoptosis is the shrinkage of the cell and its nucleus [13, 14]. Necrosis, on the other hand, is the death resulting from direct cellular injury, which is best defined by the cell and organelle swelling and lysis [14].

Notably, *C. albicans* cells show apoptotic markers with high similarity to those of mammalian cells, including phosphatidylserine externalization, reactive oxygen species accumulation, mitochondrial membrane potential dissipation, and DNA condensation and fragmentation [15]. Apoptosis is elucidated by two distinct routes, namely caspase-dependent and caspase-independent manners. According to the evidence, apoptosis-inducing factor (AIF) [16], AIF-homologous mitochondrion-associated inducer of death [17], and endonuclease G (EndoG) [18, 19] can all induce apoptotic cell death in a caspase-independent manner. With this background in mind, the present study was conducted to investigate the mechanisms of *C. glabrata* cell death caused by caspofungin. To this aim, we reported both apoptosis and necrosis in the caspofungin-treated *C. glabrata* cells.

## Materials and Methods

***Candida*** ***glabrata*** ***strain and growth conditions***

For the purpose of the study,* C. glabrata* ATCC90030 was grown on the Sabouraud dextrose agar medium (Difco, USA) and incubated at 30°C for 24 h. The strain had been previously identified by the sequencing of the complete ribosomal DNA internal transcribed spacer region.

***Antifungal susceptibility testing***

The minimum inhibitory concentration (MIC) of caspofungin was determined using the broth microdilution reference method as recommended by the Clinical and Laboratory Standards Institute (CLSI) document M27-A2 and M27-S4 [20, 21]. Caspofungin (Pfizer Central Research, Sandwich, Kent, UK) was obtained from the respective manufacturers in the form of reagent-grade powders for the preparation of the CLSI microdilution trays. To get the two times of the caspofungin concentration, it was diluted in the standard RPMI-1640 medium (Sigma Chemical Co.) buffered at a pH of 7.0 with 0.165 M morpholine-propanesulfonic acid (Sigma Chemical Co.) and L-glutamine without bicarbonate.

The antifungal agent was dispensed into 96-well microdilution trays with a final concentration of 0.016-16 µg/ml. Conidial suspensions were prepared from the isolates grown for 24 h, suspended in sterile saline solution, and adjusted by spectrophotometric measurements at a wavelength of 530 nm to a percent transmittance range of 75-77. A working suspension was made by a 1:10 dilution, followed by a 1:100 dilution of the stock suspension, with RPMI medium, which resulted in 2.5-5×10^3^ CFU/ml. In the next stage, the microdilution plates were incubated at 35°C and examined visually after 24 h; in this regard, the drug concentration that elicited 50% growth inhibition was compared with a drug-free control. The MIC values for caspofungin were compared with the CLSI (M27-A2 and M27-S4) interpretative guidelines on antifungal susceptibility testing. Briefly, *C. glabrata* with the MICs of ≤ 0.12, 0.25, and ≥ 0.5 μg/ml were considered susceptible, susceptible dose-dependent, and resistant to caspofungin, respectively. *Candida krusei* (ATCC 6258) and *C. parapsilosis* (ATCC 22019) were used as quality controls.

***Annexin V*** ***and propidium iodide staining***

The concentration of* C. glabrata* cells exposed to 1, 0.5, and 0.25 µg/ml of caspofungin was adjusted by spectrophotometric measurements at 600 nm wavelength to an absorption range of 0.2-0.3. The yeasts were then washed twice in sorbitol solution (1 M sorbitol, 0.25 m MEDTA, and 20 m MDTT), and then incubated at 30°C for 70 min in 0.01 mg/ml lyticase in 10 mM sodium citrate buffer to disrupt the cell wall. Cell apoptosis and necrosis were determined using the annexin V/propidium iodide (PI) kit according to the manufacturer’s instruction (eBioscience, USA). Following incubation with the appropriate concentrations of annexin V and PI, sample acquisition was performed using the Partec flow cytometry system. The obtained data were analyzed using the Flomax software (Partec, Germany). Nonstained cells were used as controls for background determination. For each sample, a minimum of 10,000 events were counted and then subjected to analysis. Pilot experiments were first performed to ensure that lyticase did not cause the achievement of false-positive annexin V or PI staining. All assays were performed at least in triplicate and repeated at least three times.

***DNA damage and chromatin condensation***

To investigate the features of late apoptosis in response to caspofungin, we utilized 406-diamidino-2-phenylindole dihydrochloride (DAPI) staining. The basic protocol for the DAPI staining of nuclei was carried out as previously described [22] using 1 mg of DAPI/ml (molecular probes). To this end, two drops of DAPI were applied on the sections, and then the coverslip was carefully lowered on the sections. After keeping the medium in darkness for a few minutes, DAPI stain was observed with ultraviolet excitation.

**Table 1:** Primers used for *MCA1, NUC1, *and *RDN5.8* gene expression

**Name**	**Reference gene Accession No/ identifier**	**Primer's Sequence (5’ 3’)**	**PCR product length**
*MCA1*-F *MCA1*-R	KU739085.1	AGCTCGGTTACGAAAAAGCA CGAACGTGTCTGCTGATGTT	121
*NUC1*-F *NUC1*-R	MF113057	GCGGGTTTTTCAAGTATGGA TTTCAGGAATGGCTTCATCC	192
RDN5.8-F RDN5.8-R	AB032177.1	CTTGGTTCTCGCATCGATGA GGCGCAATGTGCGTTCA	98

PCR: polymerase chain reaction

***RNA extraction and quantitative real-time reverse transcription*** ***polymerase chain reaction ***

Total RNA was extracted from *C. glabrata* strain under both caspofungin-treated and -untreated conditions. Briefly, *Candida glabrata* cells were treated with 0.25 µg/ml using a method recommended by CLSI M27-A2. However, in order to get a large mass of *C. glabrata* cells, the test was performed in 24-well trays. A positive control (i.e., untreated *C. glabrata*) was also run for each isolate in each plate. RNA extraction was performed, and the strain was grown to the mid-logarithmic phase, by using the RNAX plus kit (Sina clone, Karaj, Iran) following the instructions of the manufacturer. RNA concentrations and purity were determined by spectrophotometric measurements (Biochrom WPA Biowave II, UK). An equal amount of RNA treated with DNase was subjected to cDNA synthesis by using the PrimeScript RT reagent kit (Vivantis, Malaysia).

Primers were designed on the basis of the published sequence of the relevant genes in *C. glabrata* (Table 1). The Ribosomal 5.8s RNA gene (*RDN5.8*) was used as the endogenous reference gene. Standard curves for each gene were established with four-fold serially diluted cDNA obtained from the cells grown to the mid-logarithmic phase at 37ºC by using specific primers under appropriate PCR conditions. Real-time reverse transcription PCR was performed with the ABI Step One real-time PCR system (Applied Biosystem, USA); in addition, SYBR Premix Ex Taq II was used as a reagent specifically designed for intercalator-based real-time PCR. 

All PCR reaction mixtures contained 10 µl SYBR Premix Ex Taq II (2×), 2 µl of first strand cDNA, 0.4 µM of each primer, and dH_2_O at a final volume of 20 µl. The amplification program included an initial denaturation step at 95ºC for 30 sec, followed by 40 cycles, each of which entailed two steps performed at 95°C for 5 sec and 60°C for 30 sec. The negative controls were also included in each run. The expression of all genes was normalized to the housekeeping gene *RDN5.8s* and analyzed by means of the REST software (2009). This software uses the comparative *Ct* method (ΔΔ*Ct*) to analyze data. Experiments under each condition were performed in duplicate, and each experiment was repeated twice on two different days to assess reproducibility.

***Statistical analysis***

Statistical analysis was performed in the REST software. The software uses a pairwise fixed reallocation randomization test. The results were presented as mean and standard deviation. Differences in the results obtained at two caspofungin concentrations were assessed using the Student’s t-test. Comparison of the results at more than two caspofungin concentrations were made using ANOVA. A *P-value* less than 0.05 was considered statistically significant.

## Results

***Antifungal susceptibility testing***

The MIC for *C. glabrata* isolate was obtained as 0.5 µg/ml indicating the susceptibility of this species to caspofungin.

***Induction of Candida glabrata apoptosis and necrosis by caspofungin***

Annexin V and PI staining was performed to determine if killing by caspofungin occurred through the induction of apoptosis and/or necrosis. Annexin V and PI assays were performed on yeast spheroplasts that were prepared using lyticase, a cell wall-active lytic enzyme that hydrolyzes (1, 3)-β-D-glucan linkages. These assays showed no evidence of apoptosis or necrosis until reaching a lyticase concentration of 0.1 mg/ml (data not shown). Thereafter, the assays were performed in the presence of caspofungin using 0.02 mg/ml lyticase. 

**Figure 1:**
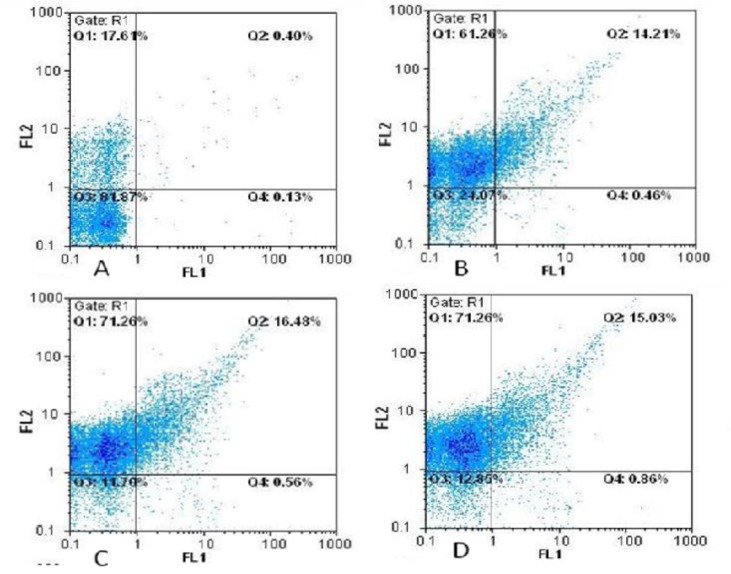
Cell apoptosis determined by annexin V-fluorescein isothiocyanate staining

**Figure 2:**
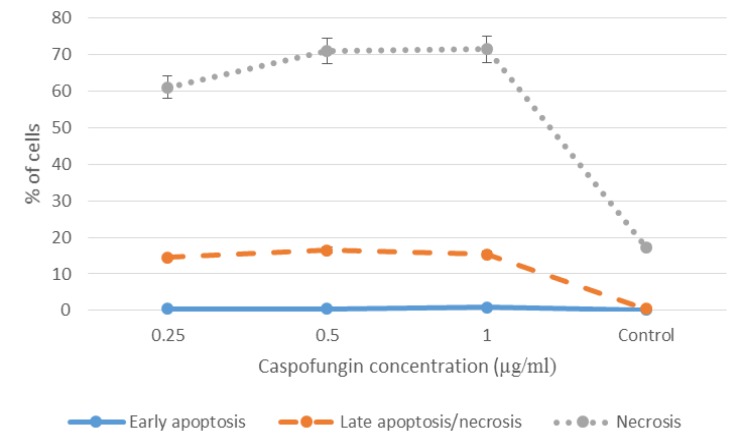
Effect of different concentrations of caspofungin on early apoptosis, late apoptosis, and necrosis

As summarized in Table 2, caspofungin induced apoptosis (early/late) in 14.67%, 17.04%, and 15.89% of the cells at 0.25, 0.5, and 1 µg/ml, respectively. On the other hand, the rate of necrosis was significantly greater than that of apoptosis following the exposure to caspofungin as observed in 71.26%, 71.26%, and 61.26% of the cells at the caspofungin concentrations of 0.25, 0.5, and 1 µg/ml, respectively (P<0.05). The results of ANOVA revealed a significant difference among the three different concentrations of caspofungin in terms of the percentage of apoptotic/necrotic cells (P<0.05; Figure 1 and Figure 2). 

***DNA damage and chromatin condensation***

**Figure 3:**
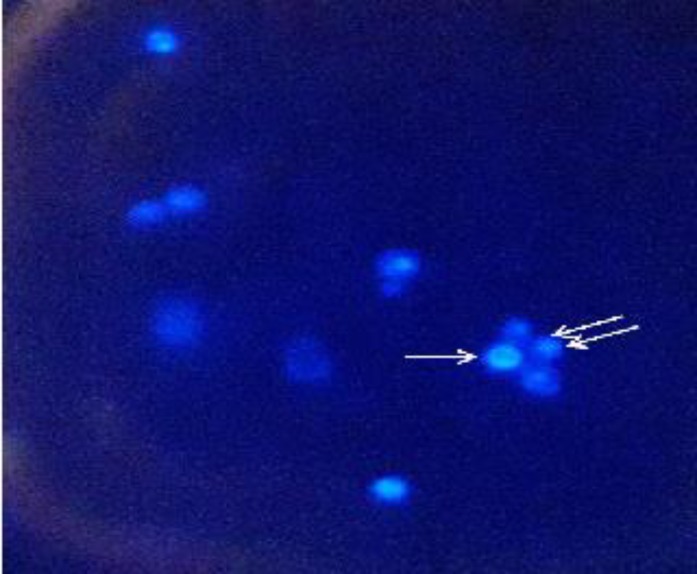
Nuclear fragmentation as demonstrated by diamidino-2-phenylindole dihydrochloride staining

Chromatin is damaged during the late stages of apoptosis due to the proteolysis of nuclear proteins, a process which results in DNA damage and chromatin condensation [23]. As depicted in Figure 3, *C. glabrata *cells exposed to caspofungin (0.125 µg/ml) had evidence of DNA damage and chromatin condensation. In this regard, the cells exposed to caspofungin exhibited irregular fragmented DNAs (single arrow), which are typical of nuclear abnormalities associated with DNA damage during apoptosis. On the other hand, regarding the control cells, DAPI staining revealed single, bright, round nucleus and peripheral cell spots corresponding to stains.

**Table 2:** Effects of caspofungin on early apoptosis, late apoptosis, and necrosis as determined by annexin V and propidium iodide staining

**Caspofungin Conc (µg/ml)**	**% of cells ** a		
	**Annexin V+/PI-** **(early apoptosis)**	**Annexin V+/PI+ (late apoptosis/necrosis)**	**Annexin V_/PI+ (necrosis)**
Control	0.13±0.006	0.4±0.03	17.61±0.4
0.25	0.46±0.05	14.21±0.7	61.26±0.5
0.5	0.56±0.2	16.48±0.4	71.26±0.1
1	0.86±0.04	15.3±0.6	71.26±0.6

a The data are presented as mean percentage±standard deviation.

(Flow cytometer analysis of the apoptotic and necrotic cells [Q1: necrotic; Q2: late apoptotic; Q3: live; Q4: early apoptotic] after 18 h of incubation with 0.25 (B), 0.5 (C) and 1(D) µg/ml caspofungin and control (A). Results are expressed as mean±standard deviation [n=3].)

(Effects of different concentrations of caspofungin on early apoptosis, late apoptosis, and necrosis, as determined by annexin V and propidium iodide staining after 18 h of incubation. Necrosis is the predominant response induced after drug exposure. Nevertheless, a higher rate of apoptosis was observed at a caspofungin concentration of 0.5 µg/ml.

(*Candida glabrata *ATCC90030 cells are shown following exposure to caspofungin at 0.125 µg/ml for 18 h (single arrow) and in the absence of caspofungin (double arrow). The cells exposed to caspofungin exhibit irregular fragmented DNAs (short arrow), which are typical of nuclear abnormalities associated with DNA damage during apoptosis. In the control cells, on the other hand, diamidino-2-phenylindole dihydrochloride staining revealed a single, bright, round nucleus and peripheral cell spots corresponding to stained mitochondria.)

**Figure 4:**
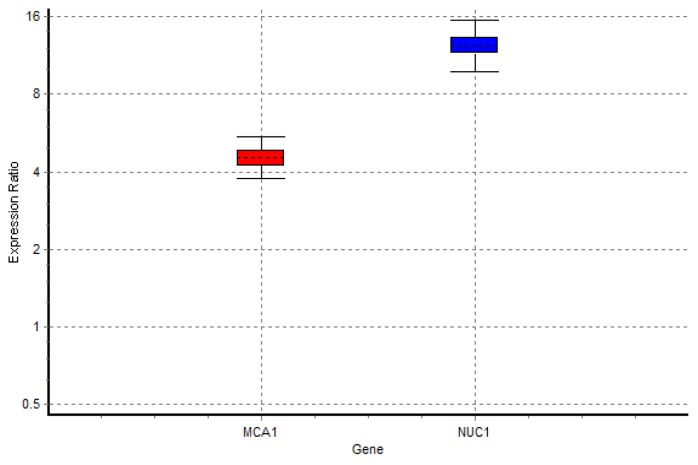
Rate of the expression of two major apoptotic genes

(*MCA1* and *NUC1* are representative of caspase-dependent and caspase-independent apoptosis induction in the yeast, respectively. Relative gene expression is the ratio of expression under caspofungin-treated condition relative to that under the untreated condition. Values of 0-1 and 1 < are respectively indicative of underexpression and overexpression when the data are normalized to the selected housekeepers. Boxes represent the interquartile range, or the middle 50% of observations. The dotted line denotes median gene expression. Whiskers signify the minimum and maximum observations.)

**Table 3:** Expression pattern of apoptosis genes in the standard isolates of *Candida glabrata*

**Gene**	**Type**	**Reaction Efficiency**	**Expression**	**Std. Error**	**95% CI**	^£^ **P(H1)**	**Result**
*RDN5.8*	bREF	1.0	1.000				
*MCA1*	aTRG	1.0	4.547	4.067 - 5.111	3.829 - 5.409	0.000	UP
*NUC1*	TRG	1.0	12.338	10.820 - 14.211	9.944 - 15.350	0.000	UP

a P(H1): Probability of alternate hypothesis pointing out that difference between the sample and control groups is only due to chance,

* TRG: target,

b REF: reference

***Quantitative real-time reverse transcription*** ***polymerase chain reaction ***

The possible effect of caspofungin on inducing expression in the two apoptotic genes (e.g., *MCA1* and *NUC*) was studied using real-time PCR assay. In addition, *RDN5.8 *gene was applied as an endogenous reference gene. Based on the results, *MCA1*, *NUC1,* and *RDN5.8s *primers had similar efficiency in a titration experiment using *C. glabrata* cDNA (1000 ng-1000 pg) in serial dilutions (data not shown). Expression of each gene was indicated as the ratio of expression relative to that of untreated logarithmic-phase-grown yeasts (Table 3). 

The REST software was employed to represent the relative expression between the treated and untreated (control) samples of all studied genes. The boxplot given by REST 2009 is a very informative way to visualize the gene expression data. It includes the smallest observation (sample minimum), lower quartile, median, upper quartile, and largest observation (sample maximum). In this boxplot, values of 0-1 and > 1 indicate underexpression and overexpression, respectively. On the basis of the REST output, the expression of the *MCA1 *and *NUC1*genes increased significantly (*P<0.05*). Figure 4 illustrates the ratio of expression under the highest concentration of caspofungin-treated condition relative to that of the untreated condition

## Discussion

As the findings of the present study indicated, caspofungin and other echinocandins can compromise *C. glabrata* cell viability via both necrosis (i.e., inhibiting cell wall integrity) and apoptosis (i.e., inducing the initiation of programmed cell death). In the present study, the evaluation of apoptosis was accomplished by the implementation of annexin V staining. This assay aims at detecting the externalization of plasma membrane phosphatidylserine, which is a critical event in the apoptotic procedure. Annexin V stains phosphatidylserine, which is a negatively charged phospholipid that is translocated from the inner leaflet of the plasma membrane to the outer part during early apoptosis [24]. 

On the other hand, since the PI stain does not permeate the cells with undamaged membranes, it facilitates the identification of the necrotic cells. Therefore, staining patterns discriminate between the live cells (annexin V), negative (annexin V-/PI-) and early apoptosis (annexin V+/PI-), necrosis (annexin V-/PI+), and late apoptosis/necrosis (annexin V+/PI+). In the present study, apoptosis was not induced within the first hour of caspofungin exposure (early caspofungin exposure). Therefore, in the present study, the incubation time of 18 h was applied to investigate the early apoptosis and late apoptosis/necrosis. The rate of late apoptosis was demonstrated by nuclear condensation (DAPI staining). 

In each of the assays, apoptosis was evident at the caspofungin concentrations lower than or equal to the MIC value (0.5 µg/mL). Moreover, the real-time assay performed on the cells exposed to 0.125 µg/mL of caspofungin revealed the overexpression of two major apoptotic genes, namely *MCA1* and *NUC1*. Caspases are the classes of cysteine-aspartic acid proteases regulated at the posttranslational level. These proteases convey a signal in a proteolytic cascade that induces apoptosis when cleaved, and lead to cell death [25]. A caspase-like protein, fitting into the type I category of metacaspases, named MCA1, was first identified in *Saccharomyces cerevisiae* [26, 27]. The recent studies demonstrated that following an apoptotic stimulus, the product of MCA1 was processed by the proteolytic removal of a 14-kDa peptide leading to the activation of metacaspase as in mammalian caspases [28]. 

 Chromatin condensation and DNA fragmentation are both the key features of apoptosis. Caspase-activated DNase is the best characterized EndoG in charge of the caspase-dependent apoptotic process [29]. *Nuc1p* has been the best-conserved yeast homologue of a mammalian apoptosis regulator to date. In a study, upon treatment with either acetic acid or H_2_O_2_, the overexpression of *EndoG* (*Nuc1p*) led to the elevation of apoptosis, compared with that in the control. In the mentioned study, this effect was reported to be depended on nuclease activity [30]. 

In *C. glabrata*, programmed cell death was characterized by the upregulation of *MCA1* and *NUC1* upon treatment with glabridin [31, 32]. According to the literature, several drugs, including ciclopiroxolamine, osmotin, pradimicin, dermaseptin, histatin [33], silver-coumarin complexes [34], farsenol [35], and amphotericin B [36], cause *C. albicans/Saccharomyces cerevisiae* cells to undergo apoptosis. Moreover, apoptosis induction was reported in the *C. albicans* exposed to caspofungin. In a study, apoptosis was induced within the first hour of caspofungin exposure. In this regard, the early apoptosis and late apoptosis/necrosis were apparent in 20-25% and 5-7% of *C. albicans *cells, respectively, following 3 h of exposure [37]. 

However, our findings revealed that most of *C. glabrata* cells were viable after 3 h of caspofungin treatment, and no sign of early apoptosis was observed in the samples. According to our results, direct toxicity due to the loss of cell wall integrity and cell lysis and the induction of programmed cell death were the responses of *C. glabrata* cells to caspofungin. However, it is well-established that nonlethal exposures to caspofungin rapidly induces the upregulation of chitin synthase genes and leads to the activation of the protein kinase C cell wall integrity signaling pathway [38]. This compensatory response is the result of cell exposure to caspofungin. Programmed cell death may be regarded as a strategy to conserve the resources for healthier cells that are more likely to survive and replicate efficiently.

## Conclusion

The findings of the present study demonstrated that caspofungin promoted both necrosis and apoptosis of *C. glabrata *cells at concentrations greater than or equal to the MIC value. Nevertheless, the real-time assay revealed that apoptosis was induced at concentrations lower than the MIC value.
